# Molecular profiling identifies prognostic markers of stage IA lung adenocarcinoma

**DOI:** 10.18632/oncotarget.20420

**Published:** 2017-08-24

**Authors:** Jie Zhang, Jinchen Shao, Lei Zhu, Ruiying Zhao, Jie Xing, Jun Wang, Xiaohui Guo, Shichun Tu, Baohui Han, Keke Yu

**Affiliations:** ^1^ Shanghai Chest Hospital, Shanghai JiaoTong University, Department of Pathology, Shanghai, China; ^2^ Tumor Initiation & Maintenance Program, Sanford Burnham Prebys Medical Discovery Institute, La Jolla; ^3^ Bioinformatics Core, Sanford Burnham Prebys Medical Discovery Institute, La Jolla; ^4^ Allele Biotechnology & Pharmaceuticals, Inc., Nancy Ridge Drive, San Diego, USA; ^5^ Shanghai Chest Hospital, Shanghai JiaoTong University, Department of Pulmonary Medicine, Shanghai, China; ^6^ Shanghai Chest Hospital, Shanghai JiaoTong University, Department of Biobank, Shanghai, China

**Keywords:** lung adenocarcinoma, gene expression profiling, differently expressed genes (DEGs), acinar, solid

## Abstract

We previously showed that different pathologic subtypes were associated with different prognostic values in patients with stage IA lung adenocarcinoma (AC). We hypothesize that differential gene expression profiles of different subtypes may be valuable factors for prognosis in stage IA lung adenocarcinoma. We performed microarray gene expression profiling on tumor tissues micro-dissected from patients with acinar and solid predominant subtypes of stage IA lung adenocarcinoma. These patients had undergone a lobectomy and mediastinal lymph node dissection at the Shanghai Chest Hospital, Shanghai, China in 2012. No patient had preoperative treatment. We performed the Gene Set Enrichment Analysis (GSEA) analysis to look for gene expression signatures associated with tumor subtypes. The histologic subtypes of all patients were classified according to the 2015 WHO lung Adenocarcinoma classification. We found that patients with the solid predominant subtype are enriched for genes involved in RNA polymerase activity as well as inactivation of the p53 pathway. Further, we identified a list of genes that may serve as prognostic markers for stage IA lung adenocarcinoma. Validation in the TCGA database shows that these genes are correlated with survival, suggesting that they are novel prognostic factors for stage IA lung adenocarcinoma. In conclusion, we have uncovered novel prognostic factors for stage IA lung adenocarcinoma using gene expression profiling in combination with histopathology subtyping.

## INTRODUCTION

Lung cancer is the leading cause of cancer related death worldwide [1] and adenocarcinoma (AC) is the most common histological type [2]. The TNM staging system is often used to determine how far the cancer has spread based on the size of the primary tumor, lymph nodes spread and metastasis [3]. Although survival of pathologic stage IA patients is expected to be much better than those at other stages, 5-year survival of these patients varies from 77% to 94.4% [4]. Stage IA patients often have very different outcomes even if undergoing the same treatment. We and others have previously reported that the new International Association for the Study of Lung Cancer/American Thoracic Society/European Respiratory Society (IASLC/ATS/ERS) lung AC classification [5] is significantly associated with patient survival, confirming the prognostic value of the new classification system [4, 6, 7]. With the recent advances of genome-wide sequencing and gene expression profiling, we have gained a deeper understanding of cancers on a molecular level. Multiple studies involving gene expression profiling of lung cancers suggest that gene expression signatures in addition to the IASLC/ATS/ERS system can be used to improve the prediction of patient survival [8–11]. In this study, we focus on identifying molecular prognostic markers that can predict the survival of stage IA lung adenocarcinoma patients, with the intention of finding reliable prognostic markers for better management and treatment of stage IA lung adenocarcinoma.

## RESULTS

### Differentially expressed genes in acinar and solid subtype lung AC

**Figure 1:**
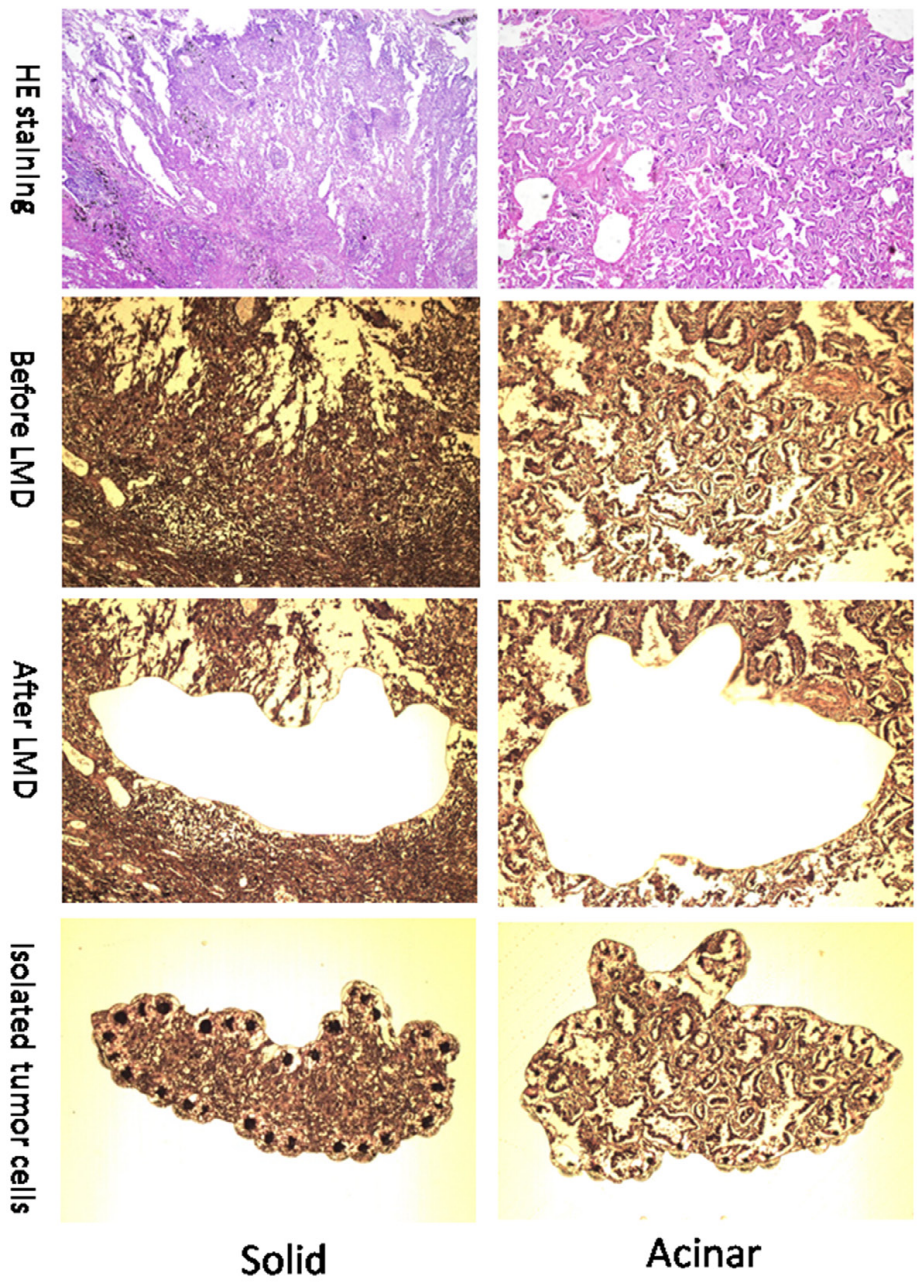
Representative images (100x) of solid and acinar subtypes of Lung AC Images in the top row show HE staining of solid (left) and acinar (right) AC. Images in the 2^nd^ row show solid (left) and acinar (right) lung tumor sections before laser microdissection (LMD). Images in the bottom two rows show sections with tumor tissues removed (3^rd^ row) and tumor fractions that were dissected out (4^th^ row).

**Figure 2:**
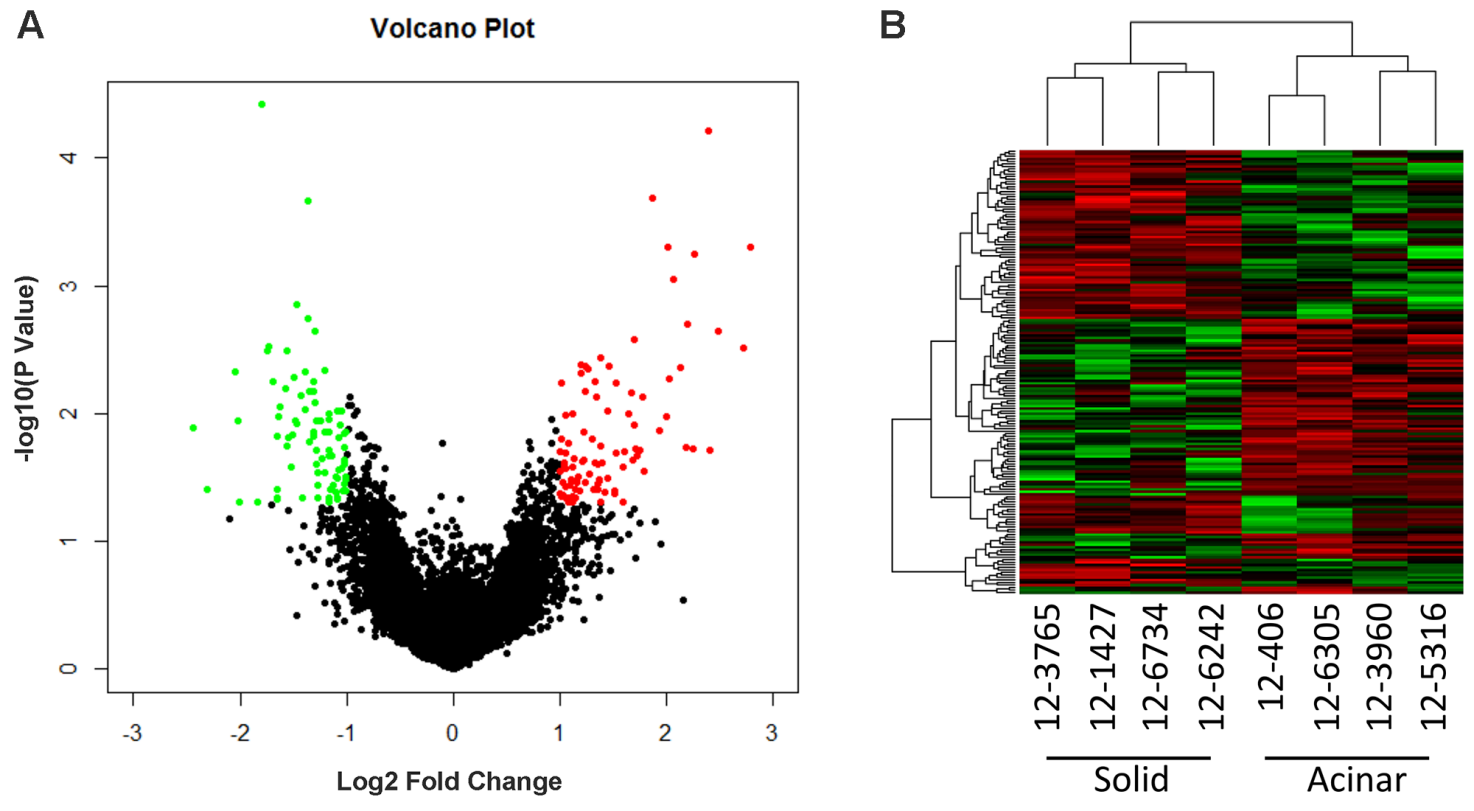
Microarray gene expression profiling of acinar and solid subtype of Lung AC **(A)** Volcano plot shows differentially expressed genes comparing acinar and solid subtype of Lung AC. Red dots highlight genes overexpressed in acinar subtype (fold change > 2, *p* value < 0.05). Green dots highlight genes underexpressed in acinar subtype (fold change > 2, *p* value < 0.05). **(B)** Heatmap shows differentially expressed genes in acinar subtype samples (12-406, 12-6305, 12-3960, 12-5316) and solid subtype samples (12-3765, 12-1427, 12-6734, 12-6242).

To identify molecular prognostic markers for stage IA lung AC, we decided to analyze the gene expression profiles of lung AC from different clinicopathological subgroups. Using tissue micro-dissection, we were able to capture at least 500 tumor cells from each patient specimen. 4 acinar subtype lung AC, which has a better prognosis, and 4 solid subtype lung AC, which has a worse prognosis, were selected for comparison of gene expression profiles using Affymetrix PrimView microarrays. Representative images of solid and acinar subtype lung AC are presented in Figure 1. Clinical information of the patients enrolled in this study such as age, clinicopathological subtype, disease-free survival (DFS) and overall survival (OS) can be found in Table 1 and Supplementary Table 1. Using a cutoff value of fold change > 2 and a *p*-value < 0.05, 521 genes were upregulated and 534 genes were downregulated in acinar subtype lung AC compared to solid subtype (Figure 2A). The heatmap of differentially expressed genes shows a clear distinction between acinar and solid subtype lung AC (Figure 2B).

**Table 1:** Clinical information of pathological stage IA lung AC patients

Patient ID	Histological Subtype	Gender	Age	DFS (Months)	OS (Months)
12-6305	Acinar	Male	43	46	46
12-3960	Acinar	Female	55	50	50
12-406	Acinar	Female	61	54	54
12-5316	Acinar	Female	52	48	48
12-6242	Solid	Male	59	10	40
12-3765	Solid	Male	62	34	50
12-1427	Solid	Female	59	53	53
12-6734	Solid	Female	60	45	45

DFS, disease free survival; OS, overall survival.

### Pathways and gene set enrichment analysis in acinar and solid subtype lung AC

**Figure 3:**
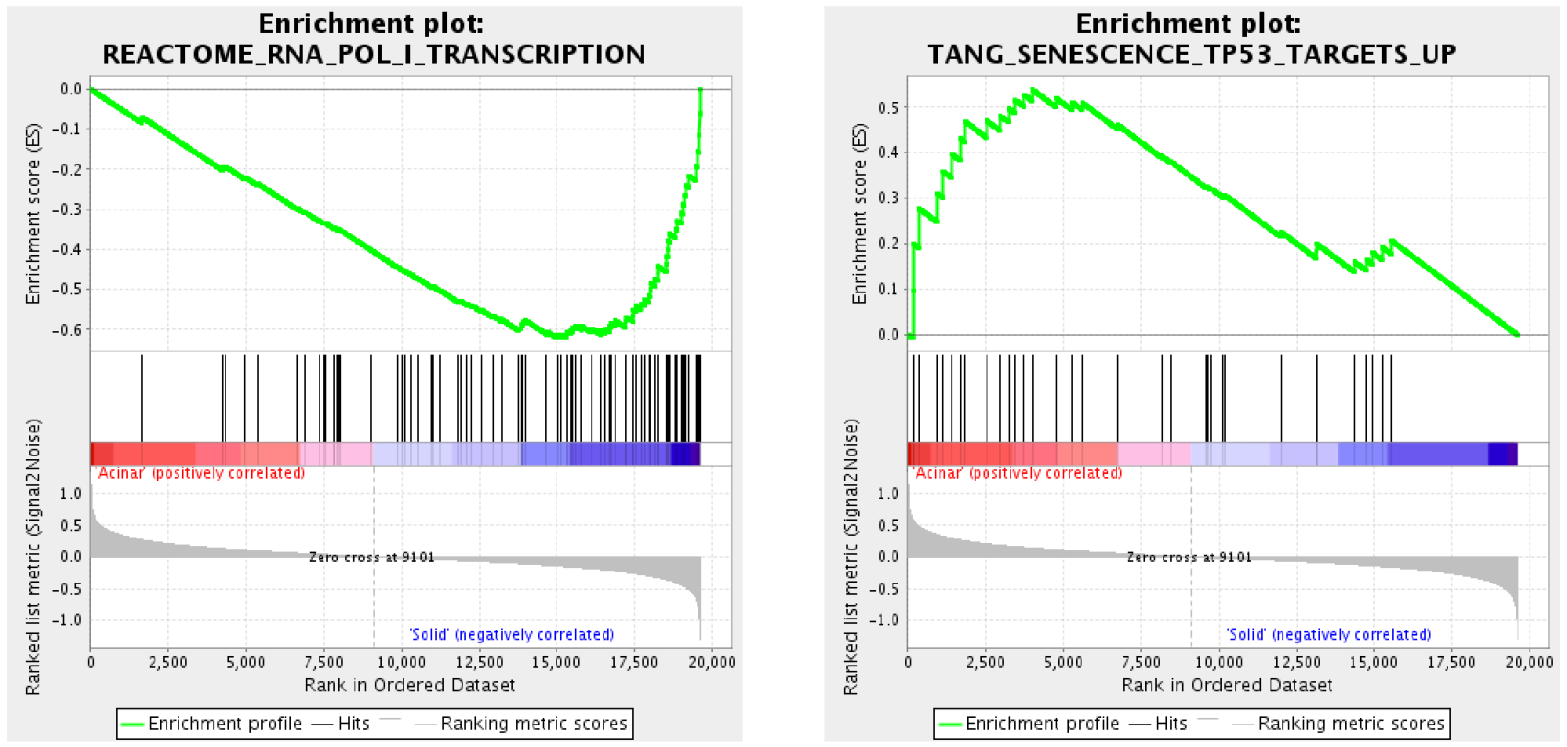
Cancer related signaling pathways are altered in different subtypes of lung AC Gene Set Enrichment Analysis (GSEA) was used to identify dysregulated signaling pathways in acinar and solid subtype of lung AC samples. Among the top dysregulated gene sets, we found enriched RNA polymerase I transcriptional activity, a common feature in human cancers, in solid subtype lung AC. Interestingly, the p53 pathway, which often regulates RNA polymerase I activity, is down-regulated in solid subtype lung AC, indicating that a worse prognosis in solid subtype lung AC is potentially due to inactivation of p53 target genes, which leads to dysregulated RNA polymerase I transcription.

Aberrations in key pathways are common in lung AC [12]. We thus used Gene Set Enrichment Analysis (GSEA) [13, 14] to identify key pathways and gene sets that are altered in acinar and solid subtypes of lung AC. 1174 gene sets were upregulated in acinar subtype, among which 9 gene sets were significant at false discovery rate (FDR) < 0.25 and 40 gene sets were significantly enriched at a nominal *p*-value < 0.01. 2691 gene sets were upregulated in solid subtype, among which 84 gene sets were significantly enriched at FDR < 0.25 and 114 gene sets were significantly enriched at a nominal *p*-value < 0.01. The top 9 enriched gene sets based on their normalized enriched score (NES) are shown for acinar subtype and solid subtype in Tables 2 and 3, respectively. Notably, the gene set that is involved in RNA polymerase I transcriptional activity is among the top gene sets enriched in solid subtype lung AC (Figure 3). Dysregulated RNA polymerase I activity is commonly associated with upregulated cell growth such as cancer [15, 16], indicating that poor prognosis of solid subtype lung AC may be due to aberrant activation of RNA polymerase I activity. Interestingly, p53 target genes were downregulated in solid subtype lung AC (Figure 3). The p53 pathway has been shown to regulate RNA polymerase I activity by repressing rRNA genes [17, 18], suggesting that dysregulated RNA polymerase I activity may be due to inactivation of the p53 pathway in solid subtype lung AC.

**Table 2:** Top 9 gene sets enriched in acinar subtype lung AC

NAME	SIZE	ES	NES	NOM p-val	FDR q-val
REACTOME_CREB_PHOSPHORYLATION_THROUGH_THE_ACTIVATION_OF_RAS	27	0.59	1.95	0	0.07
LEIN_ASTROCYTE_MARKERS	42	0.52	1.81	0	0.26
REACTOME_INWARDLY_RECTIFYING_K_CHANNELS	30	0.57	1.81	0	0.19
REACTOME_POST_NMDA_RECEPTOR_ACTIVATION_EVENTS	33	0.48	1.77	0	0.23
REACTOME_GABA_B_RECEPTOR_ACTIVATION	37	0.49	1.73	0	0.29
BILANGES_SERUM_SENSITIVE_VIA_TSC1	23	0.53	1.73	0	0.25
TANG_SENESCENCE_TP53_TARGETS_UP	33	0.54	1.73	0	0.22
REACTOME_INHIBITION_OF_INSULIN_SECRETION_BY_ADRENALINE_NORADRENALINE	24	0.55	1.73	0	0.20
REACTOME_RAS_ACTIVATION_UOPN_CA2_INFUX_THROUGH_NMDA_RECEPTOR	17	0.62	1.71	0	0.22

ES, enrichment sore; NES, normalized enrichment score; NOM p-value, nominal p value; FDR q-val, false discovery rate q value.

**Table 3:** Top 9 gene sets enriched in solid subtype lung AC

NAME	SIZE	ES	NES	NOM p-val	FDR q-val
REACTOME_TELOMERE_MAINTENANCE	75	-0.59	-1.95	0	0.16
REACTOME_RNA_POL_I_TRANSCRIPTION	82	-0.62	-1.93	0	0.13
REACTOME_RNA_POL_I_RNA_POL_III_AND_MITOCHONDRIAL_TRANSCRIPTION	114	-0.55	-1.92	0	0.11
REACTOME_PACKAGING_OF_TELOMERE_ENDS	48	-0.66	-1.89	0	0.13
DELPUECH_FOXO3_TARGETS_DN	40	-0.57	-1.88	0	0.11
KEGG_FRUCTOSE_AND_MANNOSE_METABOLISM	34	-0.51	-1.86	0	0.13
REACTOME_MEIOTIC_RECOMBINATION	81	-0.59	-1.85	0	0.12
REACTOME_RNA_POL_I_PROMOTER_OPENING	58	-0.66	-1.82	0	0.14
GALINDO_IMMUNE_RESPONSE_TO_ENTEROTOXIN	82	-0.49	-1.81	0	0.15

### Candidate genes show prognostic value in lung AC TCGA datasets

**Figure 4:**
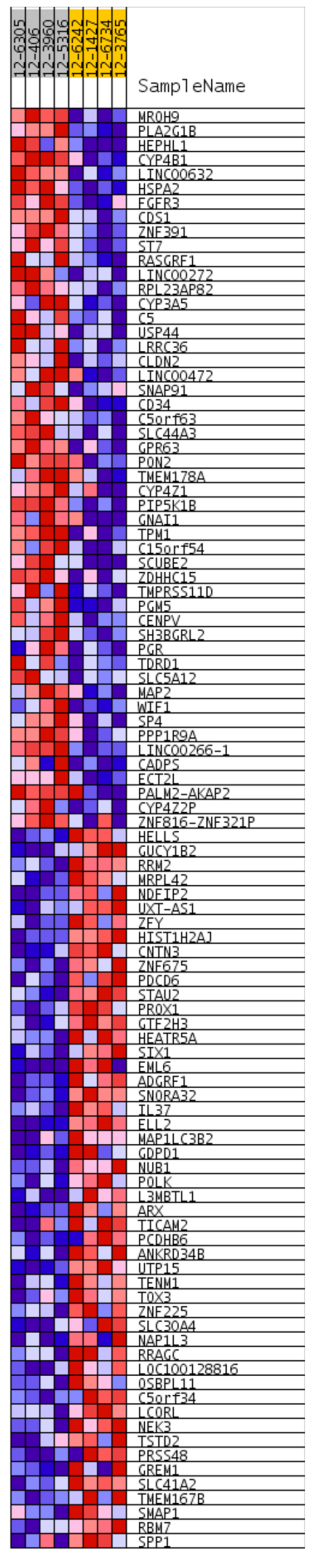
Heatmap shows top 50 differentially expressed genes between acinar and solid subtypes of lung AC Red indicates overexpression and blue indicates underexpression. Heatmap was generated in GSEA.

**Figure 5:**
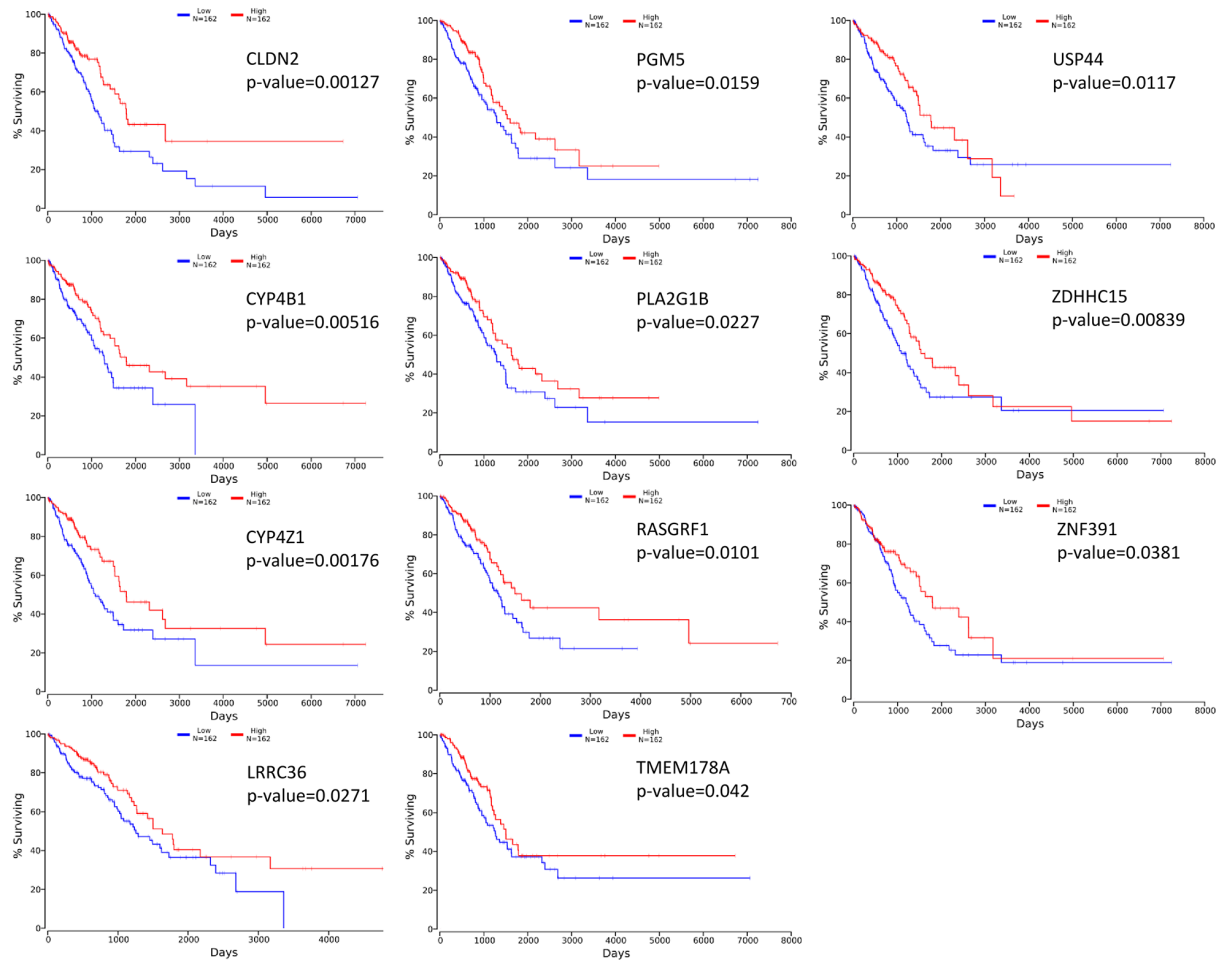
Kaplan-Meier survival curves using TCGA data validate the prognostic value of genes overexpressed in acinar subtype lung AC

**Figure 6:**
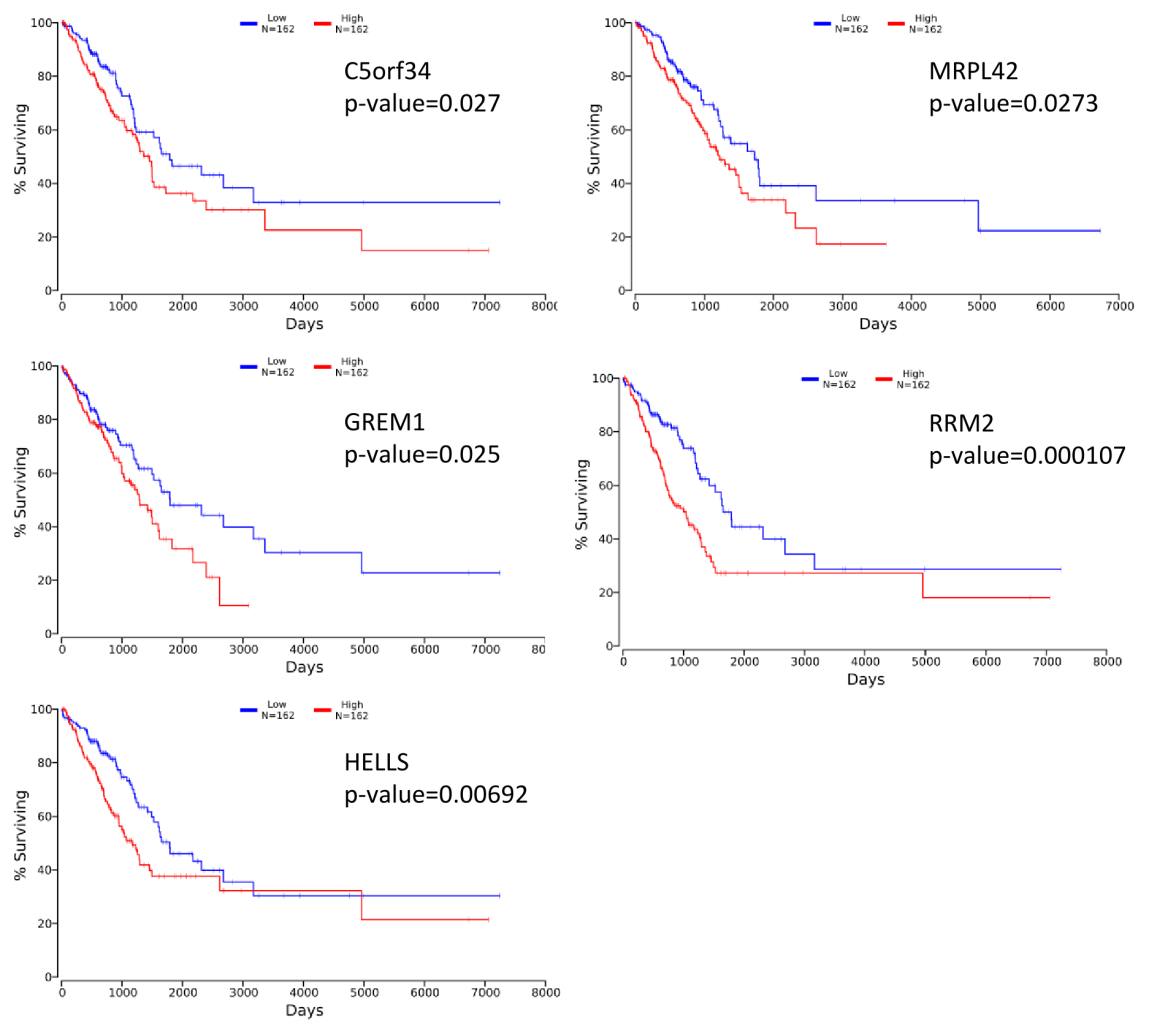
Kaplan-Meier survival curves using TCGA data validate the prognostic value of genes overexpressed in solid subtype lung AC

To evaluate if the identified prognostic markers are valuable in predicting patient survival, we focused on the top 50 differentially expressed genes between acinar and solid subtype lung AC (Figure 4). We then utilized OncoLnc [19], a tool developed for conveniently exploring survival correlations with gene expression data from 21 cancer studies performed by The Cancer Genome Atlas (TCGA). 11 genes that are overexpressed in acinar subtype lung AC show a positive correlation with patient survival. Patients with higher expression of these genes have favorable overall survival (*p*-value < 0.05) (Figure 5). 5 genes that are overexpressed in solid subtype lung AC show a negative correlation with patient survival. Patients with higher expression of these genes have worse overall survival (*p*-value < 0.05) (Figure 6).

Together, our data suggest that acinar and solid subtypes of lung AC have distinct gene expression signatures. The poor prognosis of solid subtype lung AC is potentially due to dysregulated RNA polymerase I activity and inactivation of the p53 pathway. Candidate prognostic marker genes from our study may be valuable for prediction of patient survival and guidance for disease management and treatment.

## DISCUSSION

Stage IA lung adenocarcinoma is a very heterogeneous disease. Previous studies have shown a wide range of 5-year survival and outcome from treatments for stage IA lung AC patients [20–22]. The new multidisciplinary IASLC/ATS/ERS lung AC classification system has improved the prediction of patient survival [4, 6, 7]. With the recent advances in genomics, gene expression profiling has been shown to predict the survival of patients with lung cancer accurately [23–26]. Moreover, molecular profiling may help predict patient response to treatments [27]. Several recent studies have identified gene expression signatures in non-small cell lung cancers, including lung AC, that predict patient survival. In our study, we focused on stage IA lung AC and specifically compared the gene expression signatures of specimens from 8 acinar and solid subtype lung AC patients. We identified genes that are positively correlated with survival (CLDN2, PGM5, USP44, CYP4B1, PLA2G1B, ZDHHC15, CYP4Z1, RASGRF1, ZNF391, LRRC36, TMEM178A) and negatively correlated with survival (C5orf34, MRPL42, GREM1, RRM2, HELLS). In addition, we identified gene sets and pathways that are differentially activated in acinar and solid subtype lung AC that may help predict response to treatments.

The identified genes that predict survival of stage IA lung AC patients may reveal important information for developing subtype specific therapies. CLDN2 encodes for Claudin-2, which belongs to major integral membrane proteins localized in tight junctions. CLDN2 was previously shown to be a good prognostic factor in non-small cell lung cancer [28]. PGM5 is a phosphoglucomutase involved in interconversion of glucose-1-phosphate and glucose-6-phosphage [29]. PGM5 has been found to be down-regulated in colorectal cancers [30]. USP44 is a deubiquitinase that functions as a key regulator of mitotic spindle checkpoint and loss of Usp44 in mice resulted in spontaneous tumor development, especially in lung [31]. USP44 is also frequently downregulated in human lung cancers and its decreased expression is associated with a poor prognosis [31]. CYP4B1 and CYP4Z1 are members of the cytochrome P450 monooxygenase involved in drug metabolism. It has been reported that the expression of CYP4B1 is reduced in neoplastic lung tissues compared with normal lung [32]. Mutations of the CYP4Z1 gene have been associated with progression of NSCLC [33]. GREM1 is a bone morphogenic protein (BMP) antagonist. It is overexpressed in lung AC and forced expression increases proliferation in normal lung cells [34]. RRM2 is ribonucleotide reductase regulatory subunit M2, which catalyzes the formation of deoxyribonucleotides from ribonucleotides. Multiple studies have suggested that high expression of RRM2 correlates with poor prognosis in NSCLC patients [35, 36].

In conclusion, our gene expression analysis on different subtypes of stage IA lung AC have revealed valuable prognostic factors that may help improve the prediction of patient survival. Furthermore, it may provide important implications for identifying targeted therapies in a subtype specific manner. Due to the relatively small sample size in this study, follow-up studies with a larger cohort of patient specimens will strengthen our discovery and hopefully help further improve the clinical outcomes of patients.

## MATERIALS AND METHODS

### Patient information

We used data from the records of 8 patients with pathological stage IA AC, who had undergone a lobectomy and mediastinal lymph node dissection at Shanghai Chest Hospital, Shanghai, China in 2012. Written consent was obtained from all participants and our ethics committee approved this study. None of the subjects had received radiation therapy or chemotherapy prior to surgery. Smoking history was obtained from the patients via interview. Regular follow-up evaluations after surgery for all patients included physical examination, blood examination, computed tomography scan of the chest and abdomen, brain magnetic resonance imaging, and bone scanning. Postoperative follow-up involved examination every 3 months for 2 years, every 6 months for years 3-5, and every 12 months from year 5. The median follow-up time for the entire group was 70 months (range, 10-150 months). All cases were confirmed by retrospective pathological analysis of paraffin-embedded sections.

### Microarray

At least 500 tumor cells were micro-dissected from each patient specimen. All samples were submitted to the WuXi Genome Center for microarray analysis. RNA extraction was performed followed by QC. Samples were then processed for microarray expression analysis using PrimeView Human Gene Expression Array (Thermo Fisher Scientific). Data QC was processed using Affymetrix Expression Console software (version 1.3.1). Microarray data was processed using R with the limma package. RMA normalization was applied.

### GSEA analysis

GSEA is a computational methodology to identify classes of genes that are overexpressed in a large set of genes between two biological states, such as disease phenotypes [13, 14]. This analysis was done using GenePattern (https://genepattern.broadinstitute.org/). The Molecular Signatures Database (MSigDB) C2 collection, consisting of canonical pathways and experimental signatures curated from publications, was used for the analysis.

### Survival correlation

OncoLnc (www.oncolnc.org) is a web-based database for exploring correlations between gene expression and cancer patient survival. It contains survival data for 8,647 patients from 21 cancer studies performed by The Cancer Genome Atlas (TCGA), along with RNA-SEQ expression for mRNAs and miRNAs from TCGA [19]. OncoLnc was used to link TCGA survival data to microarray gene expression data [19]. A 33% upper percentile and 33% lower percentile of expression was used to generate a Kaplan-Meier survival curve for individual genes of interest.

### Statistical analysis

For microarray analysis, the significance of differential gene expression is determined by a cut-off of p-value < 0.05 and fold change > 2. Statistics used in GSEA analysis was previously described [13]. Significance of enriched gene sets is determined by a cut-off of FDR < 0.25 and nominal p-value < 0.01. For OncoLnc, multivariate Cox regressions were used to conduct survival analysis [19] and a p-value < 0.05 is considered significant.

## SUPPLEMENTARY MATERIALS TABLES







## Abbreviations

AC: adenocarcinoma
GSEA: gene set enrichment analysis
DFS: disease free survival
OS: overall survival
TCGA: the cancer genome atlas
NES: normalized enriched score
IASLC/ATS/ERS: International Association for the Study of Lung Cancer/American Thoracic Society/European Respiratory Society
FDR: false discovery rate
