# Supplementary material for: Molecular profiling identifies prognostic markers of stage IA lung adenocarcinoma
Source: Oncotarget. 2017 Aug 24;8(43):74846–55. doi: 10.18632/oncotarget.20420 (PMC5650383; doi:10.18632/oncotarget.20420)
Supplement: Supplementary file 1 [file oncotarget-08-74846-s001.pdf]

Molecular profiling identifies prognostic markers of stage IA lung adenocarcinoma

SUPPLEMENTARY MATERIALS

Supplementary Table 1: The table of clinical variables as Supplementary about age, gender, tumor size, stage, smoking history, DFS and so on.

See Supplementary File 1

Supplementary Table 2: The list of the genes corresponding to the pathways along with the top 9 gene sets enriched in acinar subtype lung AC

NAME	GENES
REACTOME_CREB_PHOSPHORYLATION_THROUGH_THE_ACTIVATION_OF_RAS	RASGRF1,RPS6KA6,GRIN2C,GRIN2D,CALM1,ACTN2,CAMK2D,NEFL
LEIN_ASTROCYTE_MARKERS	SPARCL1,MAGI1,ATP13A5,HEPACAM,S1PR1,GLDC,CLU,MLPH,BCAN,Sep4
REACTOME_INWARDLY_RECTIFYING_K_CHANNELS	KCNJ14,KCNJ15,GABBR1,GNGT1,KCNJ6,GNG2,ABCC9,KCNJ8,GABBR2
REACTOME_POST_NMDA_RECEPTOR_ACTIVATION_EVENTS	RASGRF1,RPS6KA6,GRIN2C,GRIN2D,CALM1,ACTN2,CAMK2D,NEFL,ADCY3,CALM2,ADCY1
REACTOME_GABA_B_RECEPTOR_ACTIVATION	GNAI1,KCNJ15,ADCY6,GABBR1,GNGT1,KCNJ6,GNG2,GABBR2,ADCY9
BILANGES_SERUM_SENSITIVE_VIA_TSC1	GNAI1,MFAP4,DNAJC5G,KCTD15,GREM2,RND3
TANG_SENESCENCE_TP53_TARGETS_UP	SLC4A4,TSPAN12,WFDC1,A2M,HBG1,GABBR2,MMP3,RAPGEF4,ABCA8,HSD17B2,ALDH3A2,C7,CLIP1,SIM2
REACTOME_INHIBITION_OF_INSULIN_SECRETION_BY_ADRENALINE_NORADRENALINE	GNAI1,ADCY6,CACNB2,GNGT1,GNG2,GNG11
REACTOME_RAS_ACTIVATION_UOPN_CA2_INFUX_THROUGH_NMDA_RECEPTOR	RASGRF1,GRIN2C,GRIN2D,CALM1,ACTN2,CAMK2D,NEFL

Supplementary Table 3: The list of the genes corresponding to the pathways along with the top 9 gene sets enriched in solid subtype lung AC.

See Supplementary File 2
